# Misfit of Complete Maxillary Dentures’ Posterior Palatal Seal following Polymerisation with Four Different Autopolymerising Resins: An In Vitro Study

**DOI:** 10.3390/ma15155285

**Published:** 2022-07-30

**Authors:** Simone Schiller, Elke Rustemeier, Dominik Kraus, Helmut Stark, Frauke Müller, Karl-Heinz Utz

**Affiliations:** 1 Independent Researcher, Kastanienstraße 10, 53340 Meckenheim, Germany; simone.schiller@gmail.com; 2 Global Precipitation Climatology Centre, Deutscher Wetterdienst, Frankfurter Straße 135, 63067 Offenbach am Main, Germany; elke.rustemeier@dwd.de; 3 Department of Prosthodontics, Preclinical Education and Dental Material Science, University of Bonn, Welschnonnenstr. 17, 53111 Bonn, Germany; dominik.kraus@ukbonn.de (D.K.); helmut.stark@uni-bonn.de (H.S.); 4 Division de Gérodontologie et Prothèse Adjointe, Clinique Universitaire de Médecine Dentaire (CUMD), 1 rue Michel-Servet, 1211 Geneva, Switzerland; frauke.mueller@unige.ch; 5 Independent Researcher, Käferweg 1, 53639 Königswinter, Germany

**Keywords:** complete maxillary denture, posterior palatal seal, scraping of the master cast, misfit, autopolymerising acrylic resin, PMMA

## Abstract

Background: The majority of complete dentures are still conventionally manufactured using a flask-and-pack technique. However, the polymerization process may introduce a distortion of the denture body. The aim of this study was to evaluate the three-dimensional fit of the posterior palatal seal of maxillary complete dentures with the original impression, and to give recommendations for scraping. Methods: Four autopolymerising resins were used to manufacture 40 palatal plates each for high, medium and flat palates (total n = 120). The misfit was captured by taking a reline impression with a highly fluid silicone, the dimensions of which were measured with a flat-bed scanner. Results: The shape of the palate had a significant impact (median *p* = 0.0435), but not the resin type (median *p* = 0.2575). It was largest for the flat palate and smallest for the high palate. The largest misfit was observed in the palatal midline area (flat-palate average median: 685 µm; high and medium palates: 620 µm) decreasing towards the lateral and anterior regions. Conclusions: The results suggest compensating for the palatal misfit that occurs with autopolymerising resins by scraping a postdam of an approximately 0.7 mm depth to the master cast, decreasing towards the anterior and lateral areas. In high and medium palates, the scraping could be less pronounced.

## 1. Introduction

Retention and stability are important for the function of complete dentures and the satisfaction of patients [1]. The exact fit of the denture’s intaglio surface to the denture-bearing tissues, and the successful placement of the dental arch and denture body in the neutral zone, are of vital importance for denture function [2,3]. A good posterior palatal seal [4] seems particularly important for achieving the strong retention of maxillary dentures [5].

Three parameters ensure a good denture fit: the tight apposition of the inner part of the denture flange to the underlying tissues (inner seal), the length and volume of the functional vestibular denture flanges (peripheral seal), as well as the outer shape of the denture flanges (muscle grip) [6]. In addition, the retention of the complete denture can be enhanced by slightly compressing the underlying mucosa in the palatal vibration zone, which is also called the posterior seal [4].

With conventional flask-and-pack methods for manufacturing complete dentures, the polymerisation process may create uneven contractions of the resin, which lead to certain tensions and the warping of the denture body. The distortion patterns are largely influenced by the shape of the denture body, with a more pronounced shrinkage for areas where large volumes of resin are polymerised. However, the morphology of the palate may equally play a role [7]. Distortions become evident after deflasking, when the form of the polymerised denture no longer matches the shape of the master cast. Typically, the palatal plate slightly detaches from the cast, thereby creating a gap/misfit in the postdam area. If this discrepancy is not compensated for by scraping the posterior seal prior to denture processing, then it may clinically result in a noticeable lack of retention of the maxillary dentures [8,9,10]. Inversely, the compression of the tissues in the postdam area will improve the retention of the maxillary dentures and, hence, its clinical performance [11,12]. Ideally, the postdam should compensate for the misfit induced by the polymerisation contraction between the intaglio surface of the prosthesis and the master cast and add a slight compression to achieve suction [11]. Recommendations for the type of scraping of the postdam vary in the literature, ranging from a simple linear groove to a single or double knife-edge ridge, to a butterfly-, beach-, moustache- or even box-shaped pattern [6,12,13].

However, little is known about the influence of the morphology of the palate on the misfit of the posterior seal after polymerisation [7,13]. Further factors that influence the posterior misfit may be related to the type of resin used. If these distortion patterns were better understood, then evidence-based guidelines for scraping the master cast in the postdam area could be implemented in denture-manufacturing workflows.

This bench study investigates the null hypothesis that the misfit in the postdam area is neither different for various shapes of the palate, nor for the type of autopolymerising resin used.

## 2. Materials and Methods

From 40 patients who were treated by undergraduate students, alginate impressions of the edentulous maxilla were taken, and plaster models were obtained. From this anonymous pool of 40 patients, 3 impressions were selected, each one with flat, medium and high palatal morphologies. The three different upper jaw shapes were as follows:

- High palatal shape:

Distance from a horizontal line placed in the centre on the tuberosities in the area of the later dorsal end of the prosthesis and in the midline: 13 mm ± 0.5 mm;

- Medium palatal shape:

Distance from a horizontal line placed in the middle on the tubera in the area of the later dorsal end of the prosthesis and in the midline: 11 mm ± 0.5 mm;

- Flat palate shape:

Distance from a horizontal line placed in the middle on the tubera in the area of the later dorsal end of the prosthesis and in the midline: 7.5 mm ± 0.5 mm.

The study was approved by the Ethics Committee of the University of Bonn under reference number 231/22.

The three master casts of the flat, medium and high palates were duplicated 40 times using silicone moulds (Silflex^®^ pink, DeguDent GmbH, Bensheim, Germany) and gypsum in a mixing ratio of plaster and water, according to the manufacturer’s instructions (type 3 BonDano^®^, Wiegelmann Dental GmbH, Bonn, Germany).

Denture-like specimens without teeth were produced on the master casts using the light-polymerising resin Individo Lux^®^ (Voco GmbH, Cuxhaven, Germany). The specimens were designed according to the dimensions of the later dentures, and they consisted of a base plate of a 1.5 mm thickness. Because the complete dentures in our study were fabricated without teeth, additional material was added to the area of the alveolar ridge to simulate the dental arch. The templates were then light cured in the Luxomat^®^ D light polymerisation unit (al dente Dentalprodukte GmbH, Goslar, Germany), and the edges were smoothed.

To manufacture the specimen, a mould was needed to pour the autopolymerising resin. Therefore, two-piece silicone keys (PluLine^®^ Pluradent GmbH & Co KG, Offenbach, Germany) were fabricated with a palatal part and an outer-flange part. PluLine^®^ is a kneadable C-silicone impression material for the most precise impressions. In between these two keys, two sprue holes in the area of the second molar allowed for pouring the autopolymerising resin to fill the mould and ensure defect-free specimens. Prior to doing so, the working casts were soaked in water for 10 min and were double insulated with Aislar^®^ (Kulzer GmbH, Hanau, Germany). Two thin layers were applied with a brush to ensure the safe separation of the specimen from the cast at a later stage. After complete drying, the moulds were filled with the resin, according to the manufacturer’s instructions. Four different types of autopolymerising resins were used (Supplementary Materials); Group 1: PalaXpress^®^ (Kulzer GmbH, Hanau, Germany); Group 2: Aesthetic Autopolymerisat^®^ (Candulor AG, Glattpark, Switzerland); Group 3: ProBase^®^ Cold (Ivoclar Vivadent GmbH, Ellwangen, Germany); Group 4: FuturaGen^®^ (Schütz Dental GmbH, Rosbach, Germany) (the exact compositions of the different resins were not disclosed by the companies). With an identical protocol, 30 test dentures were fabricated for each type of resin, and, accordingly, 10 dentures each of the flat, medium and high palates, resulting in a total of 120 specimens. The polymerisation of the dentures took place in a pressure pot according to the times, temperatures and required pressures specified in the manufacturer’s instructions. After auto-polymerisation, the silicone keys and the excess resin from the sprue holes were removed, and the vestibular edges were smoothed. To ensure that the palatal length and, thus, the subsequent measuring points were identical for all dentures, the palatal border was trimmed by means of a silicone mould made on the initial specimen. Then, identical sprue holes of 1.5 mm diameters were drilled into the palatal plate, approximately 15 mm anterior to the posterior denture border, using manufactured thermoforming rails with the holes as a guide.

**Figure 1:**
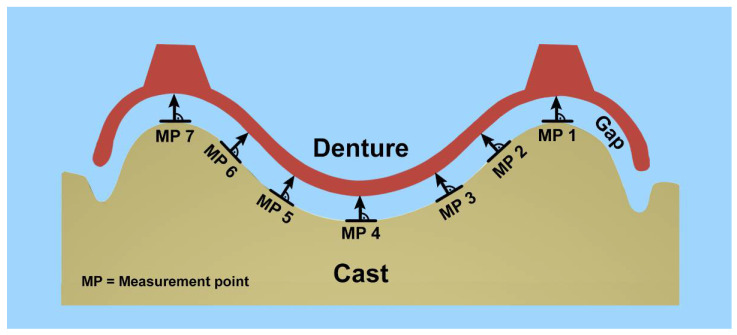
Schematic representation of the measurement points at the posterior border.

To quantify the misfit between the denture and master cast, seven measuring points (MP) were defined on the posterior border of the test specimen: one distal of each tuberosity, one in the highest point of the palate, and two further points on each side, dividing the distance between the midpoint and the tuberosities into equal thirds (Figure 1). The distance between the selected measuring points was approximately 8 mm ± 1 mm. 

**Figure 2:**
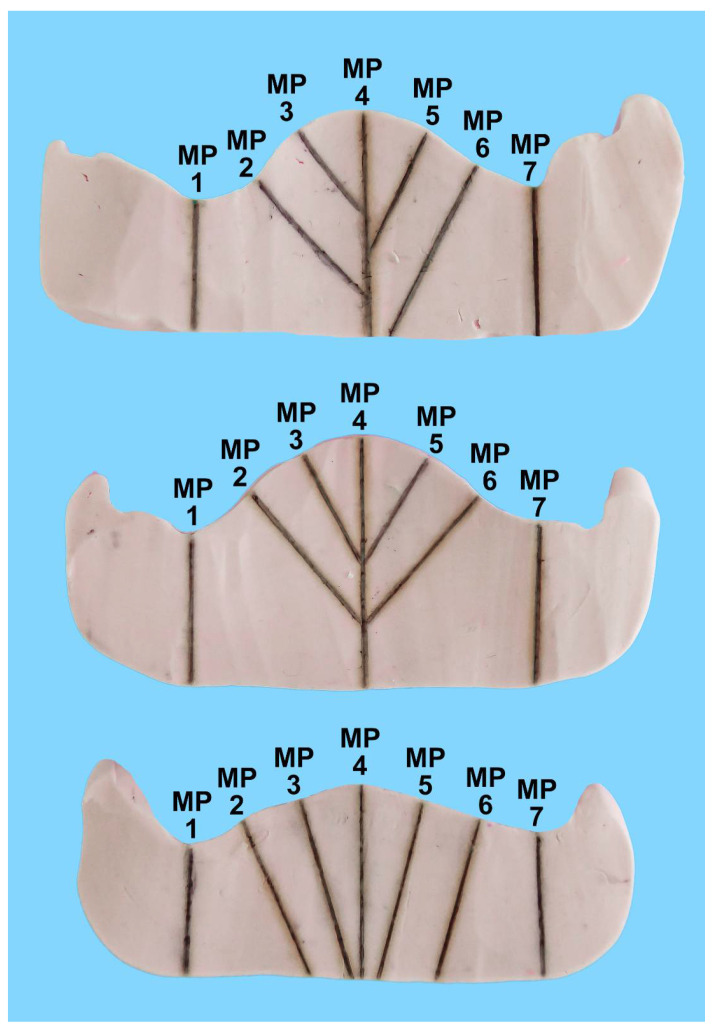
Templates for transferring the measuring points to the three samples (high, medium und flat palates). The templates were fabricated on the master casts and served to accommodate the silicone bloc with the light-body layer detached from the prosthesis (from Figure 3).

To transfer these measuring points to all specimens, three silicone keys, one for each palatal shape, were made from PluLine^®^ silicone putty (Figure 2). For this purpose, the denture templates were placed on the master casts, and the dorsal border on the casts and the measuring points were marked accordingly. Then, a silicone key was formed up to the marked measuring points. Starting from the latter, perpendicular straight lines were drawn onto the silicone key at right angles to the cast so that they could later be extended to the specimens for the interfaces.

**Figure 3:**
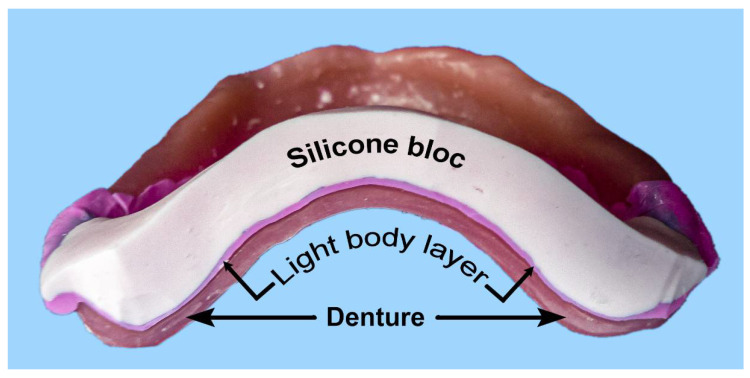
Image of the gap captured with impression material, stabilised with a silicone bloc.

The master casts were soaked in water before the dorsal misfit in the posterior area of the palatal plate was captured with the light-body silicone Plurasil^®^ (Pluradent GmbH & Co KG, Offenbach, Germany), using a reline technique with approximately 3 g of material, covering a band of a 10 mm width along the posterior palatal plate. This was a very low viscous A-silicone with high tear strength and precision, as well as excellent dimensional stability (−0.2%) and recovery after deformation (99.7%). During the setting of the silicone, the dentures were loaded with a weight of 20 N via an even support pad. Subsequently, the plaster cast was detached and replaced by adapting a PluLine^®^ silicone putty roll of a 5 mm thickness and 10 mm width to stabilise the thin layer of reline silicone, and later, the reline layer was cut with a scalpel at the measuring points, while the Plurasil^®^ light-body layer remained adhered to the dentures. The resulting sandwiches consisted of PluLine^®^ putty representing the palate, a Plurasil^®^ reline layer representing the misfit, and the resin of the test specimen (Figure 3).

Subsequently, the resin specimens were removed from the sandwich, and the template that fit exactly onto the samples was used to transfer the 7 measuring points by extending the line of the template onto the silicone wall representing the palate (Figure 2). As a last step, these templates were again replaced by a silicone bloc to sandwich and stabilise the thin reline layer of the silicone from both sides, with the marked 7 measuring points. Finally, a scalpel was used to slice the silicone blocs in the sagittal plane on the measuring points, as well as 0.5 mm parallel to the first cut, thus creating 7 slices of a 0.5 mm thickness for analysis.

These samples were scanned with a resolution of 4800 dpi (app. 5 µm) using a flat-bed scanner (CanoScanLiDE 700F, Canon, Amstelveen, The Netherlands), and they were processed using the Java image-processing program ImageJ^®^ (Rasband, W.S., ImageJ, U. S. National Institutes of Health, Bethesda, Rockville, ML, USA). Starting from the most posterior measuring points, the software determined additional points 2, 4 and 6 mm anterior, which resulted in grid with a total of 28 landmark points. For a better vision, the scanned images were enlarged on the screen. To increase the accuracy of the measurements, 5 measurements were taken from each measuring point, and their mean values and standard deviations were calculated. In total, 16,800 measurements were taken from the 120 denture specimens, providing a final 3360 readings for analysis.

In order to assess the influence of the palate shape (3 groups) and the resin material (4 groups), four analyses were performed.

**Figure 4:**
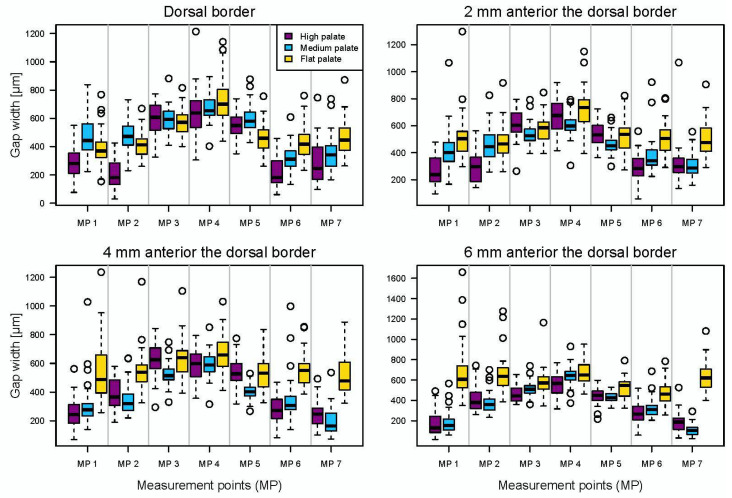
Boxplots of the misfits at the 7 measuring points for the 3 different shapes of palate (median, upper and lower quartile, minima and maxima) [18].

The median, quantiles, and thus, interquantile range, as well as the maxima and minima, are displayed with reference to the palate cross-section (Figure 4).

Because of the large number of parameters related to the palate shape, material, and measurement points (depth and width) that could be considered independently, multivariate statistics were used. Multiple linear regression (Equation (1)) [14] determined the influence of the individual parameters independent of the others. An F-test verified the levels of significance of the parameters (Table 1).

Subsequently, a Kruskal–Wallis test [15] with the null hypothesis that the groups were identical was carried out to verify whether there were significant differences. The groups included the palate shape (3 groups), material (4) and spatial parameters (width (7) and depth (4)). Therefore, a two-sided Wilcoxon rank sum test [16] was used to compare the corresponding measuring points.

Finally, the influence of the steepness of the palate (angle to the virtual occlusal plane) on the misfit was estimated using a Spearman correlation. For these analyses, areas with rather flat palates (MP 1, 4, 7), and those with a steeper inclinations to the occlusal plane (MP 2, 3, 5, 6), were grouped [17].

All statistical analyses were performed using a statistical software package (R, R Core Team (2017)). R: A language and environment for statistical computing (R Foundation for Statistical Computing, Vienna, Austria. URL: https://www.R-project.org/, version 3.4.1 (accessed on 30 June 2017). The level of significance was set at α = 0.05.

## 3. Results

The misfit at the dorsal border was found to be the largest in the midline palatal area, represented by central Measuring Point 4 (MP4). Here, it showed roughly the same gap for all three types of palate, and only the flat palate had a slightly larger gap (Figure 4) (high palate: 639 µm, inter quantile range (IQR): 190 µm; medium palate: 655 µm, IQR: 109 µm; flat palate: 701 µm, IQR: 184 µm). The misfit decreased for all types of palate forms towards the lateral areas, and it was most pronounced at the dorsal border for the high-palate group. Here, the area of the maxillary tuberosities and the upper part of the alveolar ridge of the high palate, represented by MP 1/2 and 6/7, respectively, showed significantly lower gaps than those for the medium and flat palates. The largest difference between two measuring points was found in the high-palate group at the most dorsal borders between MP 3 and 2 and MP 5 and 6, with a decrease in the gap widths of approximately 400 µm. This corresponded to the area of the steep alveolar ridges.

The misfits in the area of the measuring points anterior to the dorsal border were also largest in the midline palatal area (MP4) in all three types of palate. At 6 mm anterior, it was about 45 µm smaller than at the posterior border (Figure 4). Again, the gaps were similar for all three shapes of palate, with the high palate showing a slightly smaller gap than the medium and flat palates. From the central Measuring Point 4 towards the lateral areas, a decrease in the misfit was also found for the high and medium palates. There was a relatively continuous decrease in the gap width from the central point to the areas of the alveolar ridges 2, 4 and 6 mm anteriorly (Figure 4).

This did not apply to the flat palate, where roughly constant gap widths from the midline palatal (MP4) to the lateral areas, and particularly 4 and 6 mm anteriorly, were found (MP 1, 2, 6, 7). This indicates that, in contrast to the high and medium palates, there was no notable decrease in the gap width. This corresponded to an increase in the gap widths in the area of the tuberosities and the upper part of the flat alveolar ridge in comparison with the dorsal border (MP 1 + 2 and 6 + 7; 6 mm anterior; approximately 170 µm). However, the misfit decreased for the medium and high palates in this area by about 180 µm (MP1 + MP7 dorsal and 6 mm anterior).

In the midline palatal area (MP4), where the largest misfit was found, the gap widths ranged from 567 µm (high palate: 6 mm anterior, IQR: 159 µm) to 733 µm (flat palate: 2 mm anterior, IQR: 161 µm). The smallest gaps (107 µm medium-palate shape, 6 mm anterior to dorsal border, MP 7, IQR: 67 µm, Figure 4) were measured in the areas of the tuberosities and the upper part of the alveolar ridge (MP 1/2 and 6/7). The largest difference, with a decrease of 290 µm between the dorsal border (444 µm, IQR: 185 µm) and the measuring point 6 mm anterior (154 µm, IQR: 105 µm), was seen at the MP 1 medium-palate height.

Because the different variables were not independent, a multiple linear regression was performed to determine their individual influences (Equation (1)):

GW = [−12.0 T_0_ − 17.9 T_4_ − 33.3 T_6_ + 46.5 MP_2_ + 194.3 MP_3_ + 274.8 MP_4_ + 124.8 MP_5_ − 8.3 MP_6_ – 32.3 MP_7_ − 125.0 P_M_ − 151.4 P_H_ − 27.9 F − 44.6 P − 27.2PB + 507.0]µm (1)

F-tests were carried out to test the null hypothesis and to determine the significance and impact of the parameters on the gap width (Table 1). The parameters of the multiple linear regression for the different resin materials all showed a significant influence on the gap width (*p* < 0.05); however, the average difference between the gaps was only 44 µm.

The different palate shapes had a stronger influence on the gap width than the materials used to manufacture the specimens. Overall, the high palate had the smallest misfit, while the flat palate had the largest. In addition, the multiple linear regression showed that the high and medium palates are more similar than the flat palate. This may also be related to the fact that the selected casts of the medium and high palates are more similar in morphology than the selected shape of the flat palate. However, a larger gap variability between the measured gap dimensions within the high and medium palates than within the flat palate was found, which is related to the major differences in the morphology between the flat and steep parts in the high- and medium-palate forms. Therefore, much larger differences between the largest and smallest gaps could also be measured for the latter two palatal shapes.

The Kruskal–Wallis rank sum test evinced the existence of significantly different groups (*p* = 0.000), and a Wilcox rank sum test demonstrated that the different materials did not significantly differ from each other for the same palate type. An exception was the median across all measurement points of the flat palate for the material types FuturaGen**^®^** and PalaXPress**^®^** (median *p*-value: 0.035).

The misfit was different for the three palate types. All comparisons between the flat and high palates showed a significant difference within the same material group. Only the comparison with the medium palate was not always significant and varied in the median between a *p*-value of 0.000 when comparing flat and medium palates for the material FuturaGen**^®^**, and a *p*-value of 0.192 when comparing medium and high palates for the material Aesthetic Autopolymerisat^®^.

In addition, the measurements were analysed according to their angles with the occlusal plane using the Spearman correlations. The analyses pooled the areas with a rather flat part of the palate (MP 1, 4, 7) versus those where the slopes had steeper angulation towards the occlusal plane (MP 2, 3, 5, 6). No correlation could be established between the angle with the occlusal plane (r = −0.08).

## 4. Discussion

The results of the experiments indicate that the size of the misfit in the posterior area of a complete denture depends more on the shape of the palate than on the type of resin used. They showed different influences of the palate type and autopolymerising resins. The multiple linear regression revealed that the influence of the palate type can be significantly described by the set of parameters. This led to differences of up to 151.42 μm between the flat- and deep-palate types secured by the Wilcox rank sum test, which also demonstrated significant differences in the distributions. The results deviated for different autopolymerising resins. Again, the data were significantly described by the parameters of the multiple linear regression and led to maximum differences of 44 μm. This small difference is not relevant in clinical applications. This finding was also supported by the Wilcox rank sum test, where this difference in the gap width did not lead to significant differences in the distributions. This result is not surprising, as all four materials were mixed, moulded and polymerised according to a standard protocol.

The resins for this study and the denture-manufacturing methods employed were determined on the basis of a telephone survey that included 30 dental laboratories in the city of Bonn. Cold-curing polymers have the advantage of easier processing, shorter polymerisation times and thus a faster fabrication of the prostheses. In addition, no particular laboratory equipment is required, and the method is less costly overall. Therefore, the pour technique is still a viable alternative for clinicians and dental technicians, as the fit of the denture is similar to that of the compression-moulding and injection-moulding techniques [19]. In contrast, the heat-curing polymers show a lower residual monomer content, which, however, does not differ from that of the cold polymers after three weeks of water storage [20] (see also [21]). Regardless of the curing technique used, the presence of an unreacted residual monomer in denture-base acrylic resins is inevitable and might cause irritations and allergies [22]. Heat-curing polymers also show a higher fracture resistance and lower polymerisation shrinkage, while the stresses during the cooling phase following polymerisation are again higher. Overall, prostheses produced by the casting process with cold-cure resins do not exhibit greater inaccuracies than heat-cured ones [23]. Even the prolonged water storage of the dentures after fabrication does not significantly change the size of the dorsal gap [7,24], in contrast to prolonged dry storage, which, however, is rarely practiced by patients. Furthermore, standardised protocols for thermocycling have not yet been agreed upon [25]. The four autopolymerising resins analysed in this study were the ones most frequently used in the laboratories of the survey, which renders the results applicable to the denture manufacturing in the area.

The denture specimens were not equipped with teeth, as this would have had no effect on the outcome measure of this study, which was, namely, the posterior marginal misfit. Venus and coworkers found out that denture bases without teeth showed larger posterior gaps than dentures, but they considered them appropriate for a comparative dimensional analysis of the posterior palatal gaps [26]. In our experiments, the resin volume of the alveolar ridge was still simulated by adding resin rims and, hence, significant volume. As previous studies have reported an influence of the shape of the jaw on the posterior fit of complete dentures [7,13,27], three different shapes of palate were used in the present study.

Using a grid of 28 measuring points in the posterior area provided ample information on the pattern of the fit in the postdam area. This allowed measurements for not only the gap widths at the dorsal border, but also for the detection of their more anterior changes. This experimental setup provided a solid basis for providing clinical recommendations for scraping. Previous studies have mostly examined the dorsal misfit only at the posterior border of the palatal plate. Scanning the entire intaglio surface of the specimen and the master casts would have allowed for many more data points to be compared once the scans had been overlapped with a “closest-fit” algorithm [28]. However, these algorithms would have taken into consideration the entire intaglio surface to create the match, which might have influenced the fit in the posterior area [29]. Furthermore, substantial programming would have been necessary to analyse the misfit with regard to its inclination to the occlusal plane. Most commercially available software packages are conceived to analyse vector differences between regions of interest, rather than individual points. However, such a numerical analysis would have measured the misfit without any pressure, which eliminates the potential bias from the flow characteristics of the material and an uneven loading during its setting [30].

Unlike the clinical context, where the denture is sitting on resilient tissues, in these experiments, the reline layer was sandwiched between two nonresilient surfaces, the denture specimen and the master model, and hence, no resilience biased the results. The material and methods used in the present experiments therefore provide a robust approach to measure the posterior palatal misfit of complete dentures, given that 10 specimens were manufactured per group, and each of the 28 reference points was measured 5 times on a magnified image using the ImageJ^®^ software.

The main interest in conducting this study was to provide clinical recommendations on how to scrape a master model in the postdam area in order to compensate for the potential distortion of the denture during the resin-polymerisation process. This step is essential to achieve the retention of a maxillary complete denture and, thus, to provide the patient with a denture retention that allows normal social interactions and a good masticatory performance. The functional impairment following tooth loss and its emotional impact cannot be overestimated [31]. On the one hand, a seemingly small detail, such as scraping a postdam, may make a huge difference to the quality of life of a complete-denture wearer. On the other hand, a too pronounced scraping might displace the denture, and especially when flabby ridges are present, or it may create sore spots in the compressed areas. Moreover, a scraping of consistent thickness and width is not adequate due to the varying anatomical conditions [32]. The best results of posterior palatal scraping show “butterfly-shaped” scrapings in the area of the vibration zone, which are superior to the “single bead” or “double bead” types of scraping [12]. According to Marx (1975) [32], a butterfly-shaped scraping should be carried out, which starts narrowly at the plicae pterygomandibulares, widens to about 4 mm in the area of the glands and soft-tissue zones and narrows again towards the centre of the palate. In addition, it should slope flatly anteriorly and rise more steeply dorsally, and in no case should it be deeper than 1 mm. Based on our measurements, however, we recommend a more box-shaped scraping instead of a butterfly-shaped one. Utz [6]—before knowing the results of this study—recommended a scraping that runs to half the height of the alveolar ridges, is about 5 mm wide and, on average, about 0.5 mm deep, and is rounded in a wedge shape in the median line and dorsally. This later allows for a reduction in the denture length in cases of pressure points without immediately losing the dense attachment. Peroz et al. 1990 [7] stated that a high jaw shape requires strong scraping in the midline palatal areas and the glandular zones, but only a weak scraping on the side of the tuberae maxillae, while a flat jaw shape should have the scraping clearly applied up to the middle of the tuberae maxillae. This is also consistent with the results of our study.

**Figure 5:**
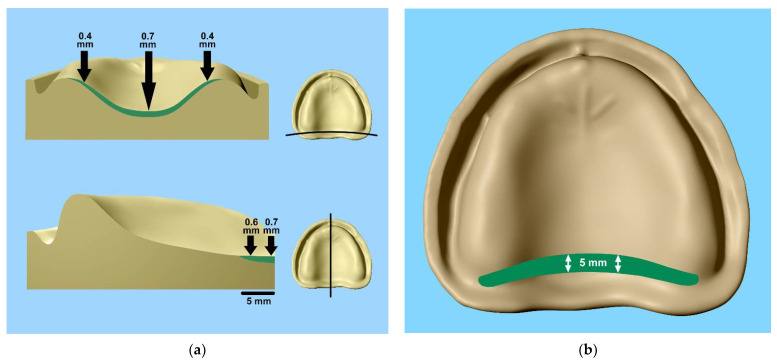
(**a**) Suggested shapes of the scraping at the tuberae maxillae at the midline palatal area (view from dorsal and sagittal); (**b**) suggested shape of the scraping at the posterior palatal seal (view from above the upper jaw cast).

Our results indicate that the misfit of the palatal plate varies according to the shape of the palate. Overall, the high-palate shapes showed the smallest inaccuracy, and the flat-palate shapes the greatest inaccuracy, of the fit of the denture bases. This is also consistent with the studies of Sykowa and Sutow (1993) [33] and Laughlin et al. (2001) [27]. However, significant differences in the palatal misfit were found between the seven most distal MPs, with larger discrepancies in the central area (MPs 3, 4 and 5), compared with the lateral parts. Whereas a similar depth of scraping can be recommended for the mid-palate for all three palatal shapes (approx. 0.7 mm), a less deep scraping is recommended in the lateral parts for high and medium palatal shapes. The scraping can be box-shaped to the mesial direction, tapering gently towards the surface of the tegument. The depth should gently fade towards the anterior, which is supported by the lower misfits measured in the 2, 4 and 6 mm measurements anterior to the posterior border. A depth of 0.7 mm would compensate for the misfit after polymerisation, and any additional depth would add to create a vacuum under the palatal plate, which is important to achieve denture retention (Figure 5). As already mentioned, the results from the present study were, in principle, confirmed by Peroz and coworkers, although they only measured the very posterior discrepancies [7].

The milling of the complete denture base from pre-polymerised pucks, as used by most current CAD/CAM technologies, is supposed to achieve better results with regard to the close fit of the dentures, as there is no more shrinkage during polymerisation [34,35,36,37]. Future research may want to investigate the palatal misfit when using industrially pre-polymerised PMMA pucks, as they are used with CAD/CAM milling techniques. These pucks are polymerised under extremely high pressure, and they therefore present a higher ultimate strength, elastic modulus and toughness than conventional denture resins [38]. Recent developments also extend denture manufacturing to 3D printing, which is also called rapid prototyping. Here, liquid resin is injected directly into the model, and hence, the fit should be even more precise. In addition, it was shown that methylmethacrylate concentrations are significantly lower in 3D-printed removable complete dentures than in the milled ones [39].

However, Srinivasan and coworkers reported a similar trueness of CAD/CAM and conventionally manufactured complete dentures in their bench experiments, all within a clinically acceptable range of 0.1 mm over the entire intaglio surface. However, the CAD/CAM specimen showed the largest variability in misfit, which might be related to the size of the milling instruments [29,40]. Other advantages of the CAD/CAM technology comprise the digital storage of the denture-manufacturing details, which allow easy replacement in case a denture has to be replaced [41]. There are many reasons why digital technology is likely to replace the traditional denture-manufacturing methods in the future [42,43,44]. Digital techniques for denture manufacturing develop at an incredible speed, and further improvements in milling techniques, as well as rapid prototyping, may be expected in the very near future [45].

## 5. Conclusions

Our in vitro study showed that the magnitude of the misfit in the area of the posterior palatal seal depends on the palatal shape and the type of resin used. Because the differences between the different autopolymerising resins were significant, but at a magnitude that has no clinical relevance, the shape of the palate seems to be of greater clinical importance. In order to maintain the suction effect in dentures, the scraping of the master cast is recommended before polymerisation. When fabricating new maxillary complete dentures with autopolymerising resins, we recommend a postdam zone in a width of about 5 mm from the dorsal margin to the anterior in a box-shaped form. In the midline palatal area, the scraping should be applied to the dorsal margin at a depth of about 0.7 mm for flat palatal shapes, tapering laterally to 0.4 mm in the direction of the tuberosities (Figure 5) and decreasing slightly anteriorly. High and medium palates require less pronounced scraping.

## Figures and Tables

**Table 1:** Gap width according to the multiple linear regression.

	Gap Width (μm)	Standard Deviation	T-Value	Significance (F-Test)
**Intercept**	507.01	9.27	54.72	0
**Depth 4 mm**	−17.94	6.77	−2.65	0.008067398
**Depth 6 mm**	−33.25	6.77	−4.91	9.37 × 10^−7^
**Depth dorsal**	−11.98	6.77	−1.77	0.076637365
**Measuring Point 2**	46.49	8.95	5.19	2.18 × 10^−7^
**Measuring Point 3**	194.26	8.95	21.7	8.14 × 10^−98^
**Measuring Point 4**	274.78	8.95	30.7	1.53 × 10^−182^
**Measuring Point 5**	124.82	8.95	13.94	5.32 × 10^−43^
**Measuring Point 6**	−8.3	8.95	−0.93	0.353597291
**Measuring Point 7**	−32.31	8.95	−3.61	0.000311736
**Medium palate**	−125.04	5.86	−21.34	8.13 × 10^−95^
**Flat palate**	−151.42	5.86	−25.84	2.13 × 10^−134^
**FuturaGen^®^**	−27.91	6.77	−4.12	3.8 × 10^−5^
**PalaXpress^®^**	−44.62	6.77	−6.59	4.97 × 10^−11^
**ProBase^®^ Cold**	−27.22	6.77	−4.02	5.88 × 10^−5^

## Supplementary Materials

The following supporting information can be downloaded at: https://www.mdpi.com/article/10.3390/ma15155285/s1.
