# Supplementary material for: Misfit of Complete Maxillary Dentures’ Posterior Palatal Seal following Polymerisation with Four Different Autopolymerising Resins: An In Vitro Study
Source: Materials (Basel). 2022 Jul 30;15(15):5285. doi: 10.3390/ma15155285 (PMC9369520; doi:10.3390/ma15155285)
Supplement: Supplementary file 1 [file materials-15-05285-s001.zip › materials-1746077-supplementary.pdf]

Information on the resins used

Product	PalaXpress® by Heraeus Kulzer	Aesthetic Autopolymerisat® by Candulor	ProBase® Cold by Ivoclar Vivadent	FuturaGen® by Schütz Dental
**Description**	Universal dental base material for dentures for injection and pouring	Cold-curing resin for the pouring and packing technique	Cold-curing resin for the pouring and packing technique	Universal denture base material for injection procedures or pouring technique
**Composition**	**cold-curing polymer** **Powder main component:** Methyl methacrylate copolymer **Liquid main components:** Methyl methacrylate, dimethacrylate cold polymer	**cold-curing polymer** **Powder:** Polymethyl methacrylate, plasticizer, benzoyl peroxide, catalyst, pigments **Liquid:** Methyl methacrylate, dimethacrylate, catalyst	**cold-curing polymer** **Powder:** Polymethyl methacrylate, plasticizer, benzoyl peroxide, catalyst, pigments **Liquid:** Methyl methacrylate, dimethacrylate, catalyst	MMA-based cold-curing resin

**Application in the pouring technique:**				
**Cast**	Isolate	hand-warm, well-watered plaster surfaces, isolate	well-watered plaster surfaces, isolate	30°C to 40°C, isolate
**Mixing ratio**	10 g powder: 7 mL liquid	15 g polymer: 10 mL monomer	15 g powder: 10 mL monomer	up to a ratio of 10: 7 free mixable
**Mixing time**	15 s	20-30 s, Let stand for 15 s	Mix well, let stand for 15 s	1 min
**Processing time at room temperature**	3 min pourable, after 4 min plastic phase from approx. 7 min	flow phase approx. 2.5-3 min, after transition phase from approx. 4 min solid, further 3 min moldable	flow phase approx. 2.5-3 min, after transition phase from approx. 4 min solid, further 3 min moldable	Mixing time 1 min
**Polymerization time**	30 min	15 min	15 min	15 min
**Water temperature**	55°C	40 °C	40 °C	45°C
**Pressure**	2 bar	2 bar	2–6 bar	2-4 bar
